# Effects of N-acetylcysteine on aging cell and obesity complications in obese adults: a randomized, double-blind clinical trial

**DOI:** 10.3389/fnut.2023.1237869

**Published:** 2023-09-19

**Authors:** Mohammad Hassan Sohouli, Ghazaleh Eslamian, Nasser Malekpour Alamdari, Maryam Abbasi, Sepideh Fazeli Taherian, Diba Behtaj, Hamid Zand

**Affiliations:** ^1^Department of Cellular and Molecular Nutrition, Faculty of Nutrition and Food Technology, Shahid Beheshti University of Medical Sciences, Tehran, Iran; ^2^Department of Cellular and Molecular Nutrition, Faculty of Nutrition and Food Technology, National Nutrition and Food Technology Research Institute, Shahid Beheshti University of Medical Sciences, Tehran, Iran; ^3^Department of General Surgery, School of Medicine, Shahid Modarres Hospital, Shahid Beheshti University of Medical Sciences, Tehran, Iran

**Keywords:** N-acetylcysteine, insulin resistance, adipose tissue, senescence, obesity

## Abstract

**Background:**

We decided to conduct this study with the aim of investigating the effects of N-Acetylcysteine (NAC) on obesity complications and senescence of visceral adipose tissue in obese adults.

**Methods and analysis:**

The present study was conducted as a randomized clinical trial (RCT) (Clinical trial registry number: IRCT20220727055563N1) on 40 obese adults candidates for bariatric surgery, who were randomly assigned to receive 600 mg of NAC (*n* = 20) or placebo as a control (*n* = 20) for 4 weeks. During bariatric surgery, visceral adipose tissue was used to examine gene expression and senescence cells using SA-β-gal.

**Results:**

Our findings showed that intervention with NAC significantly reduces SA-β-gal activity (as a marker of senescence) and expression of p16 and interleukin 6 (IL-6) genes in the visceral adipose tissue compared to placebo in obese adults for 4 weeks. In addition, our findings showed the potential and beneficial effect of NAC administration on reducing the levels of inflammatory factors including IL-6 and high-sensitivity C-reactive protein (hs-CRP), as well as the level of fasting blood sugar (FBS), homeostatic model assessment of insulin resistance (HOMA-IR), and insulin compared to placebo after adjusting for confounders. No significant effect was indicated on anthropometric factors and lipid profile.

**Conclusion:**

Findings showed that NAC, in addition to having a potential beneficial effect on reducing some of the complications caused by obesity, seems to have synolytic/senomorphic potential as well.

**Clinical trial registration:**

[https://clinicaltrials.gov/], identifier [IRCT20220727055563N1].

## Introduction

Overweight and obesity are spreading throughout the world and are recognized as a pandemic problem (1). Obesity, especially abdominal obesity, is known as a risk factor for contracting non-communicable diseases, including various cancers, diabetes mellitus, fatty liver, cardiovascular diseases, etc. (2, 3). Environmental and genetic factors as well as disturbances in energy balance and lifestyle changes have been reported as the causes of obesity (4).

Numerous studies have found a connection between obesity and higher levels of oxidative stress, which causes DNA damage (5). When DNA is broken, a series of molecules in the DNA damage response pathway are activated, which first includes the ataxia-telangiectasia-mutated (ATM) and ataxia telangiectasia and rad3-related (ATR) kinases and leads to the p53 tumor suppressor protein through CHK1 and CHK2 (6, 7). Actually, phosphorylation of p53 in N-terminal domain by this latest kinases lead to p53 activation. Depending on the type and amount of cell stress, p53 can have several responses, including growth arrest, apoptosis, or senescence (6). A natural reaction to inhibit the proliferation of damaged cells is called senescence (8). However, Senescence-associated secretory phenotype (SASP) is a major tissue function impairment caused by the increase of senescent cells (9). So that, the primary source of sterile inflammation in the adipocytes of obese people is the secretory phase of senescent cells, and this mild to moderate inflammation can also contribute to insulin resistance and other metabolic problems (7, 10). So that, inflammation and insulin resistance are among the most important mechanisms mentioned for the body’s resistance to low-calorie diets for weight loss in individuals with obesity (11). To detect senescent cells in tissues, several senescence biomarkers have been suggested that the most widely used of which relies on elevated senescence-associated β-galactosidase (SA-β-gal) (12, 13). Other markers are including p16 and p21 which relate to cell cycle arrest (14). Therefore, considering that there is a lot of evidence about the role of senescence on insulin resistance and inflammation as the main problems in obesity and related disorders and even resistance to weight loss, it seems that reduce or prevent senescence can be used as a therapeutic solution in the treatment of obesity and its complications.

Recently, the use of synolytic/senomorphic drugs and antioxidant supplements has been considered in order to investigate them on senescence activity (15). One of these drugs that has recently attracted the attention of scientists is N-acetylcysteine (NAC) (16, 17). NAC is a sulfur-containing amino acid derived from acetylated cysteine. Due to the effects of increasing antioxidant and reducing free radicals following the administration of this drug (18), the role of this drug in improving insulin secretion following the regulation of its receptor activity, reducing fat tissue, preventing and improving endothelial damage and ischemia, inhibiting phospholipid metabolism and also regulating the process of releasing pro-inflammatory cytokines has been noted (19, 20). In addition, it has been shown in a study that NAC leads to the suppresses the phosphorylation of proteins effective in the AKT/mTOR pathway as well as through the inhibition of signal transducer and activator of transcription 3 (STAT3) phosphorylation as a determining factor in the IL-6 signaling pathway, can effectively improve insulin resistance and reduce the secretion of factors effective in inflammation (21–23). In addition, a study showed that NAC, attenuated senescence and reduces kidney fibrosis through the activation of sirtuin1(SIRT1) and deacetylation of p53 (24).

Therefore, considering the importance and growing prevalence of overweight and obesity and limitations in appetite and weight control strategies for obese people, we decided to conduct this study with the aim of investigating the effects of NAC on anthropometric indices, inflammatory factors, insulin resistance, and senescence of visceral adipose tissue (using senescence-associated β-galactosidase (SAβgal) activity assay) of obese adults.

## Materials and methods

### Participants

The current study is a double-blind randomized clinical trial (RCT) study that was conducted during the years 2022–2023 and includes adults with a BMI ≥ 35 kg/m^2^ (obese) referring to Modares Hospital, Tehran, Iran. The research sample will be selected from candidates for bariatric surgery (abdominoplasty or liposuction) using convenience sampling based on inclusion and exclusion criteria. The ethics committee of the Shahid Beheshti University of Medical Sciences approved the study (IR.SBMU.NNFTRI.REC.1401.029). Moreover, this clinical trial was registered on the Iranian Registry of Clinical Trials^1^ website (IRCT20220727055563N1; registration date: 2022-08-19).

#### Inclusion and non-inclusion criteria

The following things were considered as inclusion criteria: (1) Willingness to participate and sign the informed consent form after being fully aware of the study’s aims and methodology; (2) Obese men and women aged 25–50 years and BMI ≥ 35 kg/m^2^; (3) Adults candidates for bariatric surgery (abdominoplasty or liposuction). In addition, participants who had the following conditions were prohibited from entering the study: (1) history of various inflammatory, cancer, cardiovascular, liver, diabetes, kidney, infectious, and gastrointestinal diseases, (2) use or history of use during the last 3 months of all types of supplements or drugs affecting appetite, weight, or metabolism, (3) receiving or following dietary and exercise treatments affecting weight during the last 6 months, and (4) Alcohol intake and smoking.

#### Exclusion criteria

If the studied samples experience any of the following conditions during the study, they will be excluded from the current study: (1) any event affecting the health status, (2) receiving various supplements or drugs affecting weight and metabolism despite previous warnings, (3) Failure to comply and receive medication regularly due to personal reasons or other reasons, and (4) Immigration. In addition, we checked the acceptance and compliance of the people with the desired drug after the study was completed, and if the compliance and acceptance rate was less than 80%, the people were excluded from the study.

#### Sample size calculation

The sample size for this research was determined based on the difference between the means in the serum level of tumor necrosis factor α (TNFα) as the primary outcome and in accordance with the previous research (25), which according to the type I error probability level of 5% (*α* = 0.05) and the type II error probability level of 20% (*β* = 0.20, power 80%), the number of individuals were calculated, 20 subjects in each group. Assuming 10% possible loss, 22 patients were considered in each group.

#### Study design and intervention

Forty four individuals with obesity who met the inclusion requirements for this double-blind, randomized clinical trial were randomly assigned to one of two groups that received NAC or a placebo, with the intervention lasting 4 weeks. According to the meta-analysis study conducted in this field, the doses of NAC in this field were in doses of 600–1,800 mg and the duration of the intervention was between 5 days and 12 months that most of the effect of this drug was in the dose below 1,000 and the duration of the intervention was 4–6 weeks. It has been mentioned in the studies that there is no significant difference between the use of doses of 600 to 1,000 on different factors, and even doses close to 1,000 cause a decrease in compliance in the use of the drug due to the increase in the number of times of daily drug use and finally, it may reduce the effectiveness of the drug. For this reason, we considered a dose of 600 mg for this study. For this reason, in this study, we considered a dose of 600 mg and an intervention duration of 4 weeks for the study (10). The length of the intervention period in this study was 4 weeks, and the participants of the intervention and control groups received the relevant drugs for 4 weeks before bariatrics surgery. Subjects in the intervention group received 600 mg/day of NAC, and individuals in the control group received 600 mg/day of placebo (along with lunch), which is similar in appearance and taste to the group receiving NAC and contains starch powder. The tablets were provided by Karen (placebo tablets) and Avicenna (NAC tablets) Pharmaceuticals Company. The tablets were given to the participants at the beginning of the study and they were asked to bring the empty can packages at the end of the study in fourth week to check the acceptance rate of the drugs. At the beginning of the study, all study participants received recommendations to adjust total energy intake per day based on energy intake calculated based on age, gender, and BMI. The distribution of caloric intake was estimated to include 30% fat (7% saturated and a maximum of 300 mg of cholesterol), 50% carbohydrate and 20% protein, and all subjects received the same dietary recommendations.

#### Randomization and allocation

BMI and sex were randomly assigned by stratified randomization and the permuted block randomization technique with quadruple and binary blocks to ensure the uniform distribution of these parameters in the groups. Using the web platform, the quadruple block or double block were generated based on the sample size of 44 patients.^2^

Unique codes were put on the pharmaceutical boxes to apply concealment during the randomization process, and the software also generates the desired code. No participant or researcher knows which of the two groups received the NAC or the placebo thanks to this method of concealment. The company receiving the NAC and placebos added these codes to the packaging. The medicine package including the code was given to each participant in the trial depending on the sequence that was created. The random sequence produced for the investigation was also unpredictable.

#### Primary and secondary outcomes

SA-β-gal activity test, substantial changes in the expression of the genes for tumor necrosis factor (TNF-α), IL-6, and p16, and blood concentrations of TNF-α and IL-6 were the main end measures. Weight, BMI, waist circumference (WC), FBS, insulin, HOMA-IR, total cholesterol (TC), high-density lipoprotein cholesterol (HDL-C), low-density lipoprotein cholesterol (LDL-C), triglycerides (YG), and hs-CRP were used as secondary outcome measures.

#### Anthropometric and physical activity measurements

Before and after the study, anthropometric factors were measured. Adults’ height and weight were measured with minimal clothing and without shoes. The Seca digital scale (manufactured in Germany) was used to measure each subject’s weight twice, with an accuracy of 0.01 kg. The International Physical Activity Questionnaire (IPAQ) was used to measure the amount of physical activity at the start and end of the study (26).

#### Dietary assessment

In order to check the dietary intake of patients in terms of energy, macronutrients, micronutrients and caffeine intake at the beginning and end of the research, a 24-h dietary recall questionnaire for 1 day off and 2 days off for each person (a total of 9 food note), was completed through face-to-face and telephone interviews. The analysis of dietary records was done using Nutritionist IV (N4) nutritional software.

#### Adipose tissue biopsies and SA-β-gal activity

During bariatrics surgery, paired visceral adipose tissue was harvested and using the Senescence Cells Histochemical Staining Kit (Sigma, St Louis), fresh adipose tissue samples were examined for SA-β-gal activity (27). The process was standardized as follows: At 37°C during the whole night, 100 mg of adipose tissue was incubated in 700 μL of an X-gal-containing staining solution. Fixation buffer was used to block the reaction. This method was used on human adipose tissue samples to produce a blue-green stain, which was measured on images of adipose tissue that had been digitally analyzed using Image J (NIH, United States). To determine each pixel’s cyan value, RGB photos were transformed into CMKY format.^3^ The ratio of cyan pixel intensity to the total number of pixels in the biopsy region, multiplied by 1,000, was used to calculate the SA-gal values, which were then reported as arbitrary units (AU).

#### RNA extraction, cDNA synthesis, and quantitative real-time polymerase chain reaction

Using the RNX-Plus solution and the manufacturer’s instructions (Cinaclone, Iran), total RNA was extracted from adipose tissues. Furthermore, following the manufacturer’s instructions, 1 μg of total RNA (Viragen, Iran) was used to create cDNA. Polymerase chain reaction (PCR) for TNF-α, IL-6, p16 and 18 s RNA (as an internal control) was also performed in duplicate using the following instruction and in a final volume of 20 μL: (1) 10 μL BIOFACT™ 2X Real-Time PCR Mix Master (for SYBR Green I; High. Rox, BIOFACT, South Korea); (2) 7 μL double-distilled water; (3) 0.5 μL each of forward and reverse primer (10 pmol/μL); (4) and 2 μL cDNA. Forty cycles of amplification were performed following a 15 min at 95°C initial denaturation stage. Each two step cycle included a denaturation step, 25 s at 95°C and an annealing step, 25 s at 60°C (for TNF-α an annealing step, 30 s at 60°C was used). The melt curve ranged from 60 to 95°C (StepOnePlus; Real-Time PCR, Applied Biosciences, Paisley, United Kingdom). The fold change as described by 2^−ΔΔct^ was used to obtain the results for gene expression. Supplementary Table S1 includes a list of the applied primers.

#### Biochemical measurements

Serum TC, HDL-C, and TG levels were measured using Pars Azmoon commercial kits (Tehran, Iran) by a biochemistry autoanalyzer. Serum LDL-C levels were calculated using the Friedewald Equation (28): LDL-C = TC − (HDL-C) − TG/5. FBS was determined using commercial kits from Pars Azmoon (Tehran, Iran) and serum insulin levels were determined using the immuno-turbidimetry technique. Fasting insulin (mU/L) × FBS (mmol/L)∕405 were used to compute HOMA-IR.

According to the manufacturer’s instructions, serum levels of hs-CRP were determined using a colorimetric enzyme-linked immunosorbent assay (R&D Systems, Minneapolis, MN). Furthermore, respectively, Serum TNF-α and IL-6 was assessed using ELISA kits (Crystal Day Biotech Co, Shanghai, China and Mediagnost Co, Germany).

### Statistical analysis

While qualitative factors were expressed as numbers (percentage), quantitative data were presented as mean (standard deviation). To compare the mean of the quantitative variables and the mean of their changes between the two groups at the beginning and conclusion of the research, an independent sample *t*-test was utilized. With each group, a paired sample *t*-test was employed to compare the mean of quantitative variables between before and after the intervention. The qualitative characteristics between the two groups were compared using the chi-square test or Fisher’s exact test. After taking into account potential confounding variables, the effect of NAC on quantitative variables was examined using the covariance test (ANCOVA). SPSS software version 16 was used to obtain statistical analyses, and significant levels for all tests were considered as *p*-value <0.05.

## Results

### Characteristics of the participants

**Figure 1:**
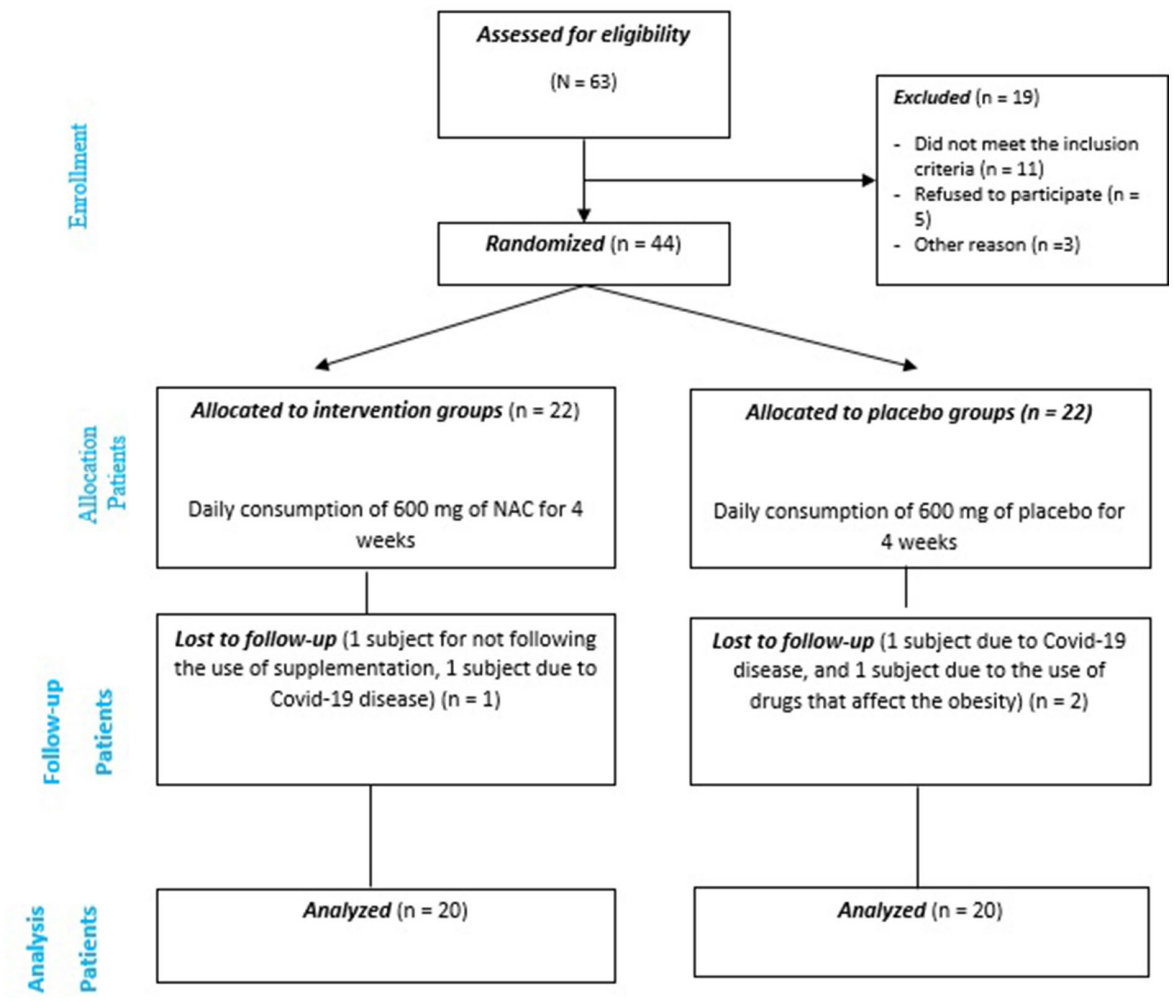
Consort flow diagram for the trial.

From 44 adult’s obesity eligible for inclusion, 40 were selected and entered the final analysis (20 in the intervention group and 20 in the placebo group) (Figure 1).

In Table 1, the individuals’ initial characteristics are listed. The participants’ mean ages were 39.68 years in the control group and 40.21 years in the intervention group. For all factors (sex, weight, BMI, WC, other metabolic characteristic, physical activity, multivitamin usage, drug use, marital status, and education level) except HOMA-IR, there was no significant difference between the two groups.

**Table 1:** Baseline characteristics of participants.

Variables		Groups, mean (SD)		*P*-value^a^
		NAC (*n* = 20)	Control (*n* = 20)	
Age, y		40.21 (5.60)	39.68 (6.91)	0.798
Female (n, %)		10 (52.6)	11 (57.9)	0.744
Weight (kg)		125.96 (13.05)	127.21 (11.90)	0.758
BMI (kg/m^2^)		44.84 (3.05)	44.31 (4.21)	0.661
Waist-circumference (cm)		137.55 (11.07)	136.27 (10.39)	0.936
FBS (mg/dL)		124.43 (38.62)	151.72 (40.06)	0.065
HOMA-IR		5.66 (1.90)	7.68 (2.45)	**0.018**
Insulin (μIU/mL)		18.79 (3.62)	20.39 (3.90)	0.206
TC (mg/dL)		156.95 (34.53)	157.07 (46.34)	0.993
TG (mg/dL)		142.99 (34.47)	165.63 (79.12)	0.276
HDL-C (mg/dL)		44.05 (4.86)	40.59 (7.91)	0.123
LDL-C (mg/dL)		90.01 (33.55)	91.82 (43.33)	0.891
IL-6 (pg/mL)		3.97 (0.44)	5.25 (2.91)	0.066
TNF-α (pg/mL)		3.55 (0.28)	3.69 (0.61)	0.367
hs-CRP (mg/dL)		0.79 (0.30)	1.09 (0.90)	0.184
Physical Activity (met.h/wk)		1170.00 (644.92)	1198.57 (749.88)	0.917
Multivitamin use (n, %)		6 (31.6)	6 (31.6)	1.00
Drug use (n, %)		8 (42.1)	10 (52.6)	0.516
Marital status (married) (n, %)		15 (78.9)	11 (57.9)	0.163
Education levels (n, %)	Less than a diploma	6 (31.6)	6 (31.6)	0.707
	Diploma	5 (26.3)	8 (42.1)	
	Bachelor	3 (15.8)	2 (10.5)	
	Higher than bachelor	5 (26.3)	3 (15.8)`	

Bold value means statistical significance of *p* < 0.05. ^a^Data obtained from independent sample *t*-test for continuous variables and Chi-square for categorical variables. NAC, N-Acetylcysteine; BMI, Body mass index; FBS, Fasting blood sugar; HDL-C, High density lipoprotein-cholesterol; HOMA_IR, Homeostatic model assessment-insulin resistance; LDL-C, Low density lipoprotein-cholesterol; TC, Total cholesterol; TG, Triglycerides; WC, Waist circumference; IL-6, Interleukin-6; TNF-α, Tumor necrosis factor-α; hs-CRP, high-sensitivity C-reactive protein.

### SAβgal activity assay (high Saβgal activity in adipose tissue samples of obese subjects)

**Figure 2:**
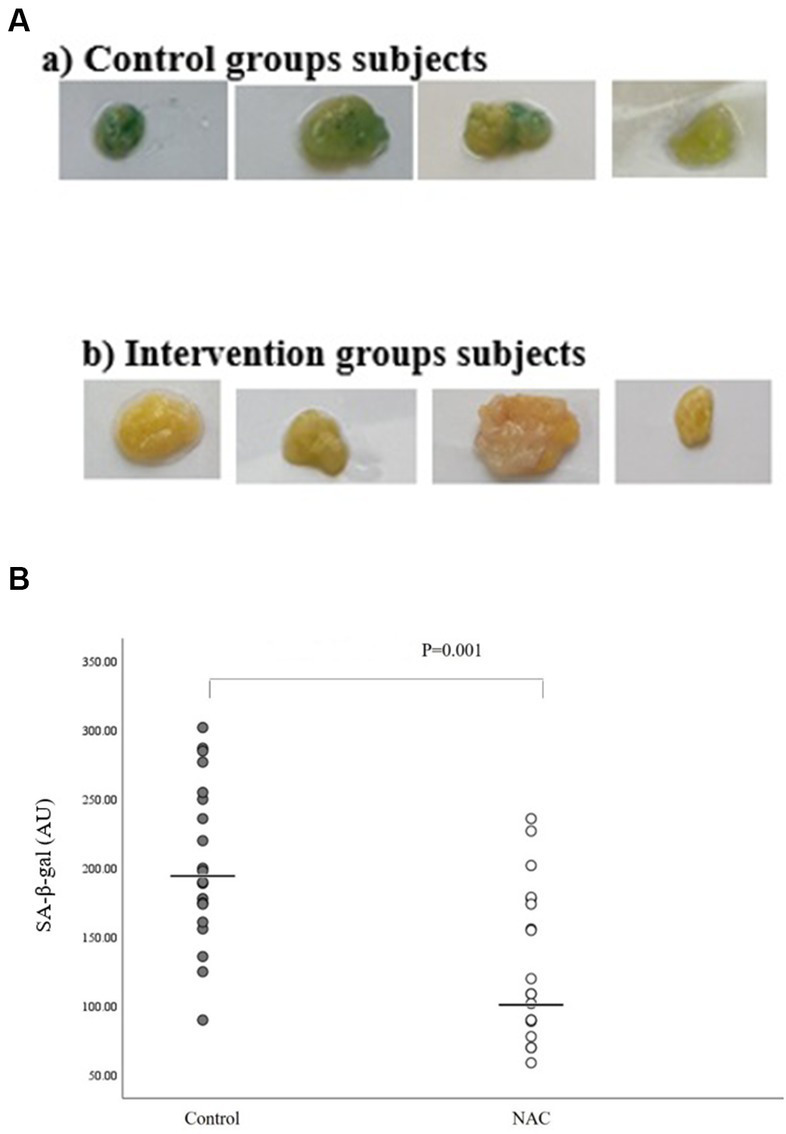
**(A)** Representative pictures of adipose tissue samples from two control and intervention groups used for quantification of SA-β-gal staining. **(B)** SA-β-gal activation quantification in intervention and control groups using an in-house quantification method (Image J).

We used a well-known cytochemical test that we optimized and standardized for human adipose tissue samples to investigate SA-β-gal activity. Individuals and fat depots both displayed very varied blue staining as a result of this assay (Figure 2A). We discovered that SA-β-gal staining and quantification were significant lower in intervention groups than in placebo group, using an in-house quantification method (Image J) (*p* = 0.001) (Figure 2B).

### Dietary intake

Dietary intake is indicated in Table 2. Table 2 lists the dietary consumption. According to the results of the 24-h food recall questionnaire and a comparison of the study’s beginning and end, in the both group, intake of vitamin E increased significantly. In addition, the mean intake of folic acid at the end of the study was significantly reduced after receiving the placebo. However, there was no significant difference in the mean consumption of calories and other nutrients between the groups at the start and end of the trial.

**Table 2:** Energy, macronutrient, and micronutrients intake at baseline and at the end of study.

	NAC			Placebo			*P*-value^b^
	Baseline	After	*P*-value^a^	Baseline	After	*P*-value^a^	
Energy (kcal/d)	3705.29 (187.18)	3679.43 (164.14)	0.066	3710.67 (183.67)	3691.59 (156.46)	0.175	0.817
Carbohydrate (g/d)	544.55 (78.88)	504.01 (71.50)	0.125	555.38 (76.09)	525.96 (72.09)	0.177	0.233
Protein (g/d)	137.50 (35.75)	137.96 (29.36)	0.670	130.95 (16.74)	129.18 (17.63)	0.169	0.174
Fat (g/d)	128.72 (30.31)	126.72 (31.96)	0.098	120.03 (29.92)	118.96 (27.76)	0.254	0.165
SFA (g/d)	40.50 (11.65)	40.31 (8.84)	0.950	40.62 (13.38)	45.74 (9.40)	0.141	0.075
MUFA (g/d)	43.28 (10.24)	41.23 (13.39)	0.507	39.85 (11.16)	47.72 (21.89)	0.141	0.278
PUFA (g/d)	28.77 (8.82)	27.31 (7.33)	0.604	26.19 (11.20)	24.89 (10.81)	0.629	0.508
Fiber (g/d)	56.11 (30.74)	56.63 (14.21)	0.952	59.93 (26.50)	47.94 (19.09)	0.122	0.121
Sodium (mg/d)	5933.60 (2496.40)	5780.27 (1786.81)	0.790	6797.52 (4676.22)	5164.25 (2299.73)	0.229	0.363
Vitamin B12 (mcg/d)	5.71 (2.94)	6.95 (3.40)	0.149	6.79 (2.57)	9.86 (7.91)	0.137	0.150
Folate (mcg/d)	659.09 (200.59)	614.37 (113.68)	0.435	789.98 (204.53)	572.07 (152.23)	**0.002**	0.338
Magnesium (mg/d)	588.84 (174.66)	553.24 (107.96)	0.482	597.95 (116.58)	532.87 (122.81)	0.148	0.591
Calcium (mg/dL)	1596.34 (418.94)	1389.14 (407.04)	0.154	1733.79 (305.62)	1673.61 (459.80)	0.631	0.058
Vitamin A (RE)	1386.65 (598.11)	1066.93 (479.83)	0.315	1099.95 (660.08)	1258.34 (856.25)	0.479	0.401
Vitamin E (mg/d)	15.52 (6.39)	24.62 (9.14)	**<0.001**	15.31 (4.15)	21.77 (6.12)	**0.003**	0.347
Vitamin C (mg/d)	266.74 (164.61)	263.39 (118.38)	0.941	260.43 (143.31)	269.74 (79.98)	0.784	0.848
Vitamin D (mcg/d)	2.34 (1.45)	2.85 (3.17)	0.481	2.91 (2.15)	2.57 (1.83)	0.268	0.471
Selenium (mg/d)	127.72 (120.93)	153.83 (57.47)	0.469	157.91 (108.65)	128.59 (48.92)	0.279	0.154
Zinc (mg/d)	18.58 (4.99)	17.33 (3.72)	0.446	18.35 (2.59)	17.15 (4.38)	0.276	0.896

Bold values means statistical significance of *p* < 0.05. ^a^Data obtained from independent sample *t*-test. Data are expressed as Mean (SD). ^a^*P*-values for comparison of within-group differences. ^b^*P*-values for comparison of mean values between two groups. PUFA, Polyunsaturated fatty acid; SFA, Saturated fatty acid; MUFA, Monounsaturated fatty acid.

### Analyses of TNF-α, IL-6, P16 gene expressions

**Figure 3:**
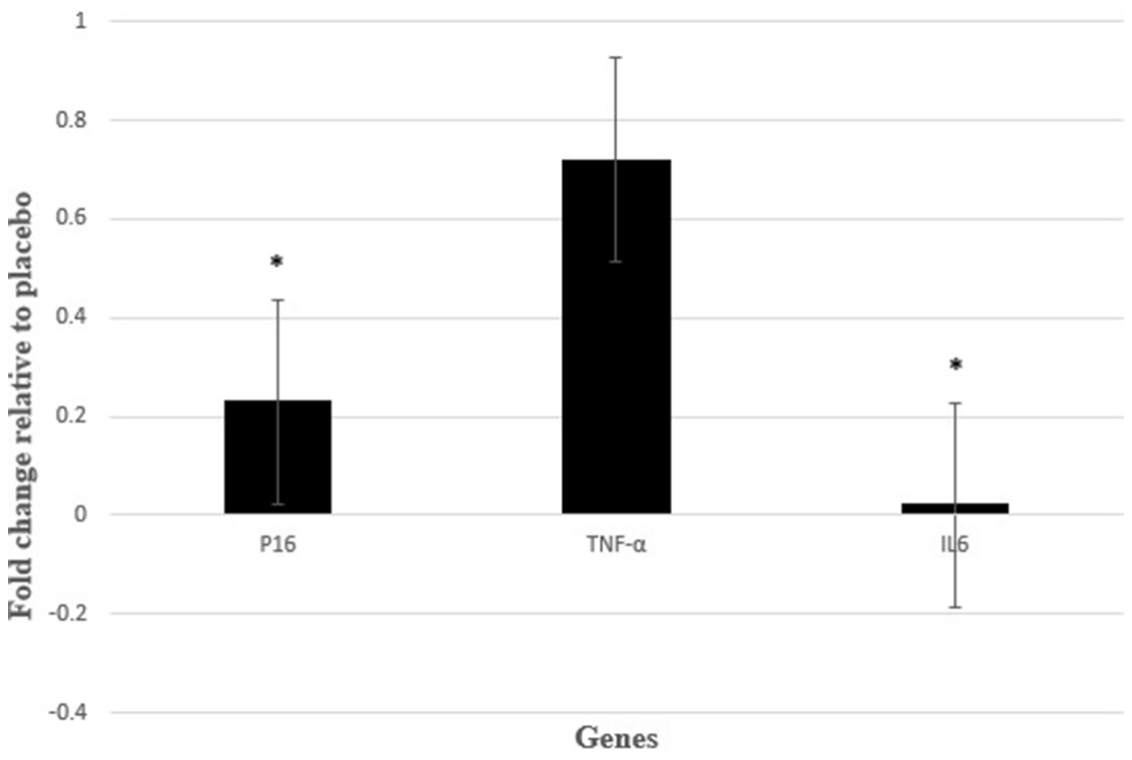
Gene expression results were expressed as the fold change defined by 2^−ΔΔCt^. Also, ΔΔCT defined by ΔCT intervention group − ΔCT control group. *P < 0.05 between control and intervention groups.

After analyzing the raw data of real-time PCR and analysis of expression fold change, our findings showed that the level of IL-6 (P 0.014; fold change: 0.02) and P16 (P 0.047; fold change: 0.23) gene expression in the intervention group with NAC was significantly lower than the control group with placebo. However, our findings did not show a significant effect on TNF-α gene expression after intervention with NAC compared to placebo. Although the expression of TNF-α gene decreased in the intervention group compared to the control group (P 0.713; fold change: 0.72) (Figure 3).

### Biochemical and anthropometry parameters

We observed significant within-group improvement for NAC and placebo group in all markers assessed. In addition, compared to the placebo group (after adjusting for baseline values, energy intake, fiber, and vitamins E, A, D, C), NAC administration for 4 weeks caused significant reduction changes for FBS, HOMA-IR, insulin, IL −6, and hs-CRP levels. However, no significant changes were observed on weight, BMI, WC, TC, TG, LDL, HDL, and TNF-α (Table 3).

**Table 3:** Anthropometric characteristics and laboratory markers at baseline and at the end of study.

	NAC				Placebo				*P*-value^b^
	Baseline	After	Change	*P*-value^a^	Baseline	After	Change	*P*-value^a^	
Weight (kg)	125.96 (13.05)	119.65 (11.82)	−6.30 (2.05)	**<0.001**	127.21 (11.90)	121.68 (11.43)	−5.53 (2.74)	**<0.001**	0.277
BMI (kg/m^2^)	44.84 (3.05)	42.16 (2.82)	−2.68 (0.59)	**<0.001**	44.31 (4.21)	41.97 (3.90)	−2.33 (0.80)	**<0.001**	0.147
WC (cm)	137.55 (11.07)	127.83 (9.88)	−9.71 (3.11)	**<0.001**	137.27 (10.39)	128.86 (10.09)	−8.40 (2.13)	**<0.001**	0.141
FBS (mg/dL)	124.43 (38.62)	111.92 (34.83)	−12.51 (6.02)	**<0.001**	151.72 (40.06)	144.47 (39.50)	−7.25 (3.69)	**<0.001**	**<0.001**
HOMA-IR	5.66 (1.90)	3.55 (1.04)	−2.82 (0.86)	**<0.001**	7.68 (2.45)	6.04 (1.89)	−1.63 (0.90)	**<0.001**	**<0.001**
Insulin (μIU/mL)	18.79 (3.62)	13.13 (3.27)	−5.66 (2.62)	**<0.001**	20.39 (3.90)	16.83 (2.60)	−3.55 (2.11)	**<0.001**	**<0.001**
TC (mg/dL)	156.95 (34.53)	149.94 (33.58)	−7.01 (2.82)	**<0.001**	157.07 (46.34)	150.66 (44.41)	−6.40 (3.36)	**<0.001**	0.528
TG (mg/dL)	142.99 (34.47)	132.16 (33.72)	−10.83 (4.49)	**<0.001**	165.63 (79.12)	158.41 (73.22)	−7.22 (7.99)	**0.002**	0.107
HDL-C (mg/dL)	44.05 (4.86)	43.01 (4.71)	−1.04 (1.08)	**0.001**	40.59 (7.91)	38.72 (8.47)	−1.86 (1.56)	**<0.001**	0.173
LDL-C (mg/dL)	90.01 (33.55)	85.77 (32.31)	−4.23 (3.74)	**<0.001**	91.82 (43.33)	88.34 (40.79)	−3.48 (4.56)	**0.006**	0.565
IL-6 (pg/mL)	3.97 (0.44)	3.33 (0.46)	−0.63 (0.27)	**<0.001**	5.25 (2.91)	4.90 (2.84)	−0.34 (0.12)	**<0.001**	**<0.001**
TNF-α (pg/mL)	3.55 (0.28)	3.26 (0.26)	−0.29 (0.07)	**<0.001**	3.69 (0.61)	3.44 (0.62)	−0.25 (0.06)	**<0.001**	0.079
hs-CRP (mg/dL)	0.79 (0.30)	0.36 (0.27)	−0.42 (0.09)	**<0.001**	1.09 (0.90)	0.82 (0.85)	−0.26 (0.11)	**<0.001**	**<0.001**

Bold values means statistical significance of *p* < 0.05. Data are expressed as Mean (SD). ^a^P-values for comparison of within-group differences. ^b^P-value changes for between-group differences using analyses of covariance, considering baseline values, energy intake, fiber, and vitamin E, A, D, C as covariate. NAC, N-Acetylcysteine; BMI, Body mass index; FBS, Fasting blood sugar; HDL-C, High density lipoprotein-cholesterol; HOMA_IR, Homeostatic model assessment-insulin resistance; LDL-C, Low density lipoprotein-cholesterol; TC, Total cholesterol; TG, Triglycerides; WC, Waist circumference; IL-6, Interleukin-6; TNF-α, Tumor necrosis factor- α; hs-CRP, high-sensitivity C-reactive protein.

## Discussion

One of the common features of obesity and aging is the accumulation of adipose tissue that has undergone senescence (6). Although cellular senescence is a defensive mechanism that inhibits tumor growth, when it occurs in adipose tissue, it results in impaired adipogenesis, inflammation, abnormal adipocytokine secretion, and insulin resistance, all of which contribute to adipose dysfunction (6). Therefore, reducing or preventing senescence activity in adipose tissue, in addition to being useful in the treatment of obesity along with other treatment methods, can prevent the occurrence of many metabolic and inflammatory problems. For this reason, in this study, we used adipose tissue to identify senescent cells using the SA-β-gal biomarker. Our findings also demonstrated the significant effect of NAC on reducing senescence activity using a marker known as SA-β-gal, both quantitatively and qualitatively. This finding of ours was confirmed when we observed that the expression of p16 gene was significantly decreased. Recently, in line with our study, the use of synolytic drugs and antioxidant supplements has been considered in order to investigate them on senescence activity. So that in a study published on diabetic kidney patients in 2019, the findings indicated a reduction in the senescence activity of adipose tissue following the use of the combination of dasatinib (as a synolytic drug) and quercetin (as an antioxidant supplement) within 11 days through the reduction of cells expressing p16, p21, and SA-β-gal activity (12). In addition, circulating SASP factors including IL-6 were decreased. In another study, the mentioned drug combination (Dasatinib and Quercetin) significantly decreased the expression of pro-inflammatory cytokines and the level of SASP factors in the circulation (29). However, no comprehensive human study has been conducted to evaluate the effect of NAC on senescence. The results of a study examining the impact of NAC (500 mg/kg) on cisplatin-induced senescence in the kidney tissues of male C57BL/6 mice treated with cisplatin showed that NAC therapy decreased the positive region of SA-Gal staining and downregulated the mRNA levels of SASP factors (IL-6, IL-1, and TNF-), as well as the p53 and p21 proteins. These findings suggest that NAC is a potent inhibitor of cisplatin-induced kidney senescence (24). This study also showed that N-acetylcysteine attenuates cisplatin-induced senescence in a SIRT1 activation-dependent manner. According to the evidence, the Nicotinamide adenine dinucleotide (NAD)-dependent deacetylase SIRT1 is crucial for both cellular senescence and organismal aging (30). In another study, NAC enhanced cognitive and motor abilities while reducing age-related memory loss and behavioral alterations in the senescence-accelerated prone 8 (SAMP8) mouse model (31). Furthermore, it has been suggested that NAC reduces the markers indicating the level of oxidative DNA damage and the frequency of DNA deletion, and in this way, it deals with genetic instability as one of the factors that increase the level of senescence and its related problems (21). However, the potential beneficial effect of this drug on reducing the level of inflammatory factors through antioxidative and cytoprotective activities of per- and polysulfides can also be one of the other discussed mechanisms (21). An increase in the level of inflammatory factors is one of the factors affecting DNA damage.

According to the results of the present study, NAC administration decreased IL-6 and hs-CRP concentration compared to placebo administration. On the other hand, it was observed that this drug significantly reduces the expression of IL-6 genes compared to placebo. Although there was no significant difference between the two groups regarding TNF-α (level of serum and expression gene). Examining and aligning the serum level and gene expression of these two inflammatory factors can have an increasing effect on confirming the results. In line with our findings, in a meta-analysis study in 2020 that included 24 RCT studies and 1,057 participants, the findings showed that oral administration of NAC (with a variable dose of 400 to 2000 mg/day) significantly reduced serum levels of CRP and IL-6. While there was no significant effect on TNF-α following oral intake of this drug (18). However, injecting this drug caused a significant decrease in TNF-α. The conflicting results on TNF-α following oral intake and injection of NAC administration may be due to the fact that NAC injection increases its bioavailability (32). Oral administration appears to have a poor bioavailability (10%), since oral administration of 600–1,200 mg of NAC per day only produced plasma NAC concentrations of 16 and 35 M, respectively. This may be a result of the slow intestinal absorption rate and/or the rapid first-pass metabolism (32). This reason can also explain the lack of meaningful reporting of our findings on NAC.

In addition, it was reported in our study that NAC administration improves insulin resistance and insulin secretion compared to placebo. However, in the present study, no significant findings were observed on lipid factors after receiving NAC compared to placebo. Following intake of NAC in humans, various effects on glycemic markers and level of lipid profile have been documented. In line with our results, an RCT study reported that receiving 1800 mg of NAC for 12 weeks significantly reduced all glycemic factors, including fasting glucose and insulin, as well as improved insulin resistance (33). However, it did not report a significant effect on lipid profile. According to the research of Javanmanesh et al., in compare to metformin, receiving NAC at a dose of 1800 mg per day for 6 months decreased the level of FBS, insulin, HOMA-IR, and serum TG in women with polycystic ovary syndrome (PCOS), but it had no effect on their other lipid profiles (34). Furthermore, one study reported that co-administration of NAC and metformin for 12 months in patients with nonalcoholic steatohepatitis (NASH) decreased serum FBS, fasting insulin and insulin resistance, HDL-C, and TG levels but did not change serum concentrations of TC (35). However, it is unclear in this study (Oliveira et al.) whether the observed potential beneficial effect are due to the effects of NAC or metformin. In general, through regulating the gene expression of peroxisome proliferator-activated receptors gamma (PPAR-γ), inhibiting enhancer binding protein beta, and modifying the PI3K/Akt insulin signaling pathway, NAC may have an impact on glucose and lipoprotein metabolism (19).

Furthermore, compared to the control group, no significant effect was observed on anthropometric factors after receiving NAC. The results are in line with a meta-analysis article published in 2020. In this meta-analysis (36), which included 7 RCT articles, the findings showed that NAC administration has no significant effect on weight, BMI and waist circumference. We hypothesized that administration of NAC, an antioxidant that contains sulfur, may aid in weight reduction in these individuals by regulating energy-related genes and hormones including leptin and adiponectin, as well as lowering insulin resistance and the anti-inflammatory pathway (36). In fact, inflammation has been linked strongly to obesity (37). However, it is possible that antioxidant drugs or supplements reduce inflammation by some other mechanism rather than affecting adipokines and obesity (38).

Changes in the consumption of vitamin E and folic acid during our study can be due to various reasons, such as recommendations for adjusted energy intake and proper distribution of macronutrients throughout the day by the researchers of this study.

The main strength of this article is the design and novelty of the RCT, given that this research was the first human study to investigate the effects of NAC administration on senescence and factors associated with metabolic disorders in obese subjects. In addition, conducting this study on visceral adipose tissue is another strength of this study. However, there are limits to our investigations that offer viewpoints. First, we could not assess obesity-related hormones and body fat percentage due to budget constraints. Second, senescence in adipose tissue is evaluated globally by the SA-gal test. As suggested by Biran et al. (39), more research is required to clearly identify the many senescent cell types that are present in adipose tissue by combining the labeling of senescence markers identity. Also, considering that RNA expression is not always equal to protein expression and may affect the results of evaluated inflammatory markers, this point can be another limitation of our study. Failure to perform immunohistochemistry (IHC) as the gold standard for evaluating inflammatory changes in fat tissue, as well as the short duration of the intervention, which can affect the results of the present study, can be limitations of this study.

## Conclusion

The findings of the present study showed that NAC significantly reduces the activity of SA-β-gal as well as the expression of p16 and IL-6 genes in the visceral adipose tissue of obese adults compared to placebo. Also, after adjusting for confounders, reducing and potential beneficial effect on inflammatory factors such as IL-6 and hs-CRP, as well as glucose metabolism were observed after receiving NAC. Although the purpose of this study was mostly focused on reducing the complications caused by obesity, according to our findings, it seems that this drug can have synolytic/senomorphic potential as well.

## Data availability statement

The raw data supporting the conclusions of this article will be made available by the authors, without undue reservation.

## Ethics statement

The studies involving humans were approved by the Shahid Beheshti University of Medical Sciences. The studies were conducted in accordance with the local legislation and institutional requirements. The participants provided their written informed consent to participate in this study.

## Author contributions

GE, HZ, and MS contributed to the conception, design, and statistical analysis. MS, GE, NM, MA, SF, DB, and HZ contributed to the data collection and manuscript drafting. MS and HZ supervised the study. All authors approved the final version of the manuscript.

## Conflict of interest

The authors declare that the research was conducted in the absence of any commercial or financial relationships that could be construed as a potential conflict of interest.

## Publisher’s note

All claims expressed in this article are solely those of the authors and do not necessarily represent those of their affiliated organizations, or those of the publisher, the editors and the reviewers. Any product that may be evaluated in this article, or claim that may be made by its manufacturer, is not guaranteed or endorsed by the publisher.

## Footnotes

^1^ www.irct.ir

^2^ www.sealedenvelope.com

^3^ https://imagej.net/RGB_to_CMYK

## References

[1] Blüher M. Obesity: global epidemiology and pathogenesis. Nat Rev Endocrinol. (2019) 15:288–98. doi: 10.1038/s41574-019-0176-8 30814686

[2] Dhawan D Sharma S. Abdominal obesity, adipokines and non-communicable diseases. J Steroid Biochem Mol Biol. (2020) 203:105737. doi: 10.1016/j.jsbmb.2020.105737, PMID: 32818561 PMC7431389

[3] Prospective Studies Collaboration Whitlock G Lewington S Sherliker P Clarke R Emberson J. Body-mass index and cause-specific mortality in 900 000 adults: collaborative analyses of 57 prospective studies. Lancet. (2009) 373:1083–96. doi: 10.1016/S0140-6736(09)60318-4, PMID: 19299006 PMC2662372

[4] Heymsfield SB Wadden TA. Mechanisms, pathophysiology, and management of obesity. N Engl J Med. (2017) 376:254–66. doi: 10.1056/NEJMra1514009 28099824

[5] Włodarczyk M Nowicka G. Obesity, DNA damage, and development of obesity-related diseases. Int J Mol Sci. (2019) 20:1146. doi: 10.3390/ijms20051146, PMID: 30845725 PMC6429223

[6] Liu Z Wu KK Jiang X Xu A Cheng KK. The role of adipose tissue senescence in obesity-and ageing-related metabolic disorders. Clin Sci. (2020) 134:315–30. doi: 10.1042/CS20190966, PMID: 31998947

[7] Palmer AK Xu M Zhu Y Pirtskhalava T Weivoda MM Hachfeld CM. Targeting senescent cells alleviates obesity-induced metabolic dysfunction. Aging Cell. (2019) 18:e12950. doi: 10.1111/acel.12950, PMID: 30907060 PMC6516193

[8] Sun Y Coppé J-P Lam EW-F. Cellular senescence: the sought or the unwanted? Trends Mol Med. (2018) 24:871–85. doi: 10.1016/j.molmed.2018.08.002 30153969

[9] Özcan S Alessio N Acar MB Mert E Omerli F Peluso G. Unbiased analysis of senescence associated secretory phenotype (SASP) to identify common components following different genotoxic stresses. Aging. (2016) 8:1316–29. doi: 10.18632/aging.100971, PMID: 27288264 PMC4993333

[10] Hirosumi J Tuncman G Chang L Görgün CZ Uysal KT Maeda K. A central role for JNK in obesity and insulin resistance. Nature. (2002) 420:333–6. doi: 10.1038/nature01137 12447443

[11] Van Baak MA Mariman EC. Mechanisms of weight regain after weight loss—the role of adipose tissue. Nat Rev Endocrinol. (2019) 15:274–87. doi: 10.1038/s41574-018-0148-4, PMID: 30655624

[12] Hickson LJ Prata LGL Bobart SA Evans TK Giorgadze N Hashmi SK. Senolytics decrease senescent cells in humans: preliminary report from a clinical trial of Dasatinib plus quercetin in individuals with diabetic kidney disease. EBioMedicine. (2019) 47:446–56. doi: 10.1016/j.ebiom.2019.08.069, PMID: 31542391 PMC6796530

[13] Dimri G Lee X Basile G Acosta M Scott G Roskelley C. A biomarker that identifies senescent human cells in culture and in aging skin *in vivo*. Proc Natl Acad Sci U S A. (1995) 92:9363–7. doi: 10.1073/pnas.92.20.9363 7568133 PMC40985

[14] Gorgoulis V Adams PD Alimonti A Bennett DC Bischof O Bishop C. Cellular senescence: defining a path forward. Cells. (2019) 179:813–27. doi: 10.1016/j.cell.2019.10.005 31675495

[15] Xu M Pirtskhalava T Farr JN Weigand BM Palmer AK Weivoda MM. Senolytics improve physical function and increase lifespan in old age. Nat Med. (2018) 24:1246–56. doi: 10.1038/s41591-018-0092-9, PMID: 29988130 PMC6082705

[16] Kulkarni AS Aleksic S Berger DM Sierra F Kuchel GA Barzilai N. Geroscience-guided repurposing of FDA-approved drugs to target aging: a proposed process and prioritization. Aging Cell. (2022) 21:e13596. doi: 10.1111/acel.13596, PMID: 35343051 PMC9009114

[17] Kumar P Liu C Hsu JW Chacko S Minard C Jahoor F. Glycine and N-acetylcysteine (GlyNAC) supplementation in older adults improves glutathione deficiency, oxidative stress, mitochondrial dysfunction, inflammation, insulin resistance, endothelial dysfunction, genotoxicity, muscle strength, and cognition: results of a pilot clinical trial. Clin Transl Med. (2021) 11:e372. doi: 10.1002/ctm2.372, PMID: 33783984 PMC8002905

[18] Askari M Faryabi R Mozaffari H Mofrad MD. The effects of N-acetylcysteine on serum level of inflammatory biomarkers in adults. Findings from a systematic review and meta-analysis of randomized clinical trials. Cytokine. (2020) 135:155239. doi: 10.1016/j.cyto.2020.155239, PMID: 32799012

[19] Fulghesu AM Ciampelli M Muzj G Belosi C Selvaggi L Ayala GF. N-acetyl-cysteine treatment improves insulin sensitivity in women with polycystic ovary syndrome. Fertil Steril. (2002) 77:1128–35. doi: 10.1016/S0015-0282(02)03133-3, PMID: 12057717

[20] Elnashar A Fahmy M Mansour A Ibrahim K. N-acetyl cysteine vs. metformin in treatment of clomiphene citrate–resistant polycystic ovary syndrome: a prospective randomized controlled study. Fertil Steril. (2007) 88:406–9. doi: 10.1016/j.fertnstert.2006.11.173, PMID: 17335818

[21] Reliene R Fischer E Schiestl RH. Effect of N-acetyl cysteine on oxidative DNA damage and the frequency of DNA deletions in atm-deficient mice. Cancer Res. (2004) 64:5148–53. doi: 10.1158/0008-5472.CAN-04-0442, PMID: 15289318

[22] Mahapatra SK Bhattacharjee S Chakraborty SP Majumdar S Roy S. Alteration of immune functions and Th1/Th2 cytokine balance in nicotine-induced murine macrophages: immunomodulatory role of eugenol and N-acetylcysteine. Int Immunopharmacol. (2011) 11:485–95. doi: 10.1016/j.intimp.2010.12.020, PMID: 21237301

[23] Michailidis Y Karagounis LG Terzis G Jamurtas AZ Spengos K Tsoukas D. Thiol-based antioxidant supplementation alters human skeletal muscle signaling and attenuates its inflammatory response and recovery after intense eccentric exercise. Am J Clin Nutr. (2013) 98:233–45. doi: 10.3945/ajcn.112.049163, PMID: 23719546

[24] Li C Xie N Li Y Liu C Hou FF Wang J. N-acetylcysteine ameliorates cisplatin-induced renal senescence and renal interstitial fibrosis through sirtuin1 activation and p53 deacetylation. Free Radic Biol Med. (2019) 130:512–27. doi: 10.1016/j.freeradbiomed.2018.11.006 30447351

[25] Zhang Q Ju Y Ma Y Wang T. N-acetylcysteine improves oxidative stress and inflammatory response in patients with community acquired pneumonia: a randomized controlled trial. Medicine. (2018) 97:e13087. doi: 10.1097/MD.0000000000013087 30407312 PMC6250560

[26] Hallal PC Victora CG. Reliability and validity of the international physical activity questionnaire (IPAQ). Med Sci Sports Exerc. (2004) 36:556. doi: 10.1249/01.MSS.0000117161.66394.07 15076800

[27] Rouault C Marcelin G Adriouch S Rose C Genser L Ambrosini M. Senescence-associated β-galactosidase in subcutaneous adipose tissue associates with altered glycaemic status and truncal fat in severe obesity. Diabetologia. (2021) 64:240–54. doi: 10.1007/s00125-020-05307-0, PMID: 33125520

[28] Friedewald WT Levy RI Fredrickson DS. Estimation of the concentration of low-density lipoprotein cholesterol in plasma, without use of the preparative ultracentrifuge. Clin Chem. (1972) 18:499–502. doi: 10.1093/clinchem/18.6.499, PMID: 4337382

[29] Justice JN Nambiar AM Tchkonia T LeBrasseur NK Pascual R Hashmi SK. Senolytics in idiopathic pulmonary fibrosis: results from a first-in-human, open-label, pilot study. EBioMedicine. (2019) 40:554–63. doi: 10.1016/j.ebiom.2018.12.052, PMID: 30616998 PMC6412088

[30] Hwang J-w Yao H Caito S Sundar IK Rahman I. Redox regulation of SIRT1 in inflammation and cellular senescence. Free Radic Biol Med. (2013) 61:95–110. doi: 10.1016/j.freeradbiomed.2013.03.015, PMID: 23542362 PMC3762912

[31] Marie A Meunier J Brun E Malmstrom S Baudoux V Flaszka E. N-acetylcysteine treatment reduces age-related hearing loss and memory impairment in the senescence-accelerated prone 8 (SAMP8) mouse model. Aging Dis. (2018) 9:664–73. doi: 10.14336/AD.2017.0930, PMID: 30090654 PMC6065287

[32] Borgström L Kågedal B Paulsen O. Pharmacokinetics of N-acetylcysteine in man. Eur J Clin Pharmacol. (1986) 31:217–22. doi: 10.1007/BF00606662 3803419

[33] Panahi Y Ostadmohammadi V Raygan F Sharif MR Sahebkar A. The effects of N-acetylcysteine administration on metabolic status and serum adiponectin levels in patients with metabolic syndrome: a randomized, double-blind, placebo-controlled trial. J Funct Foods. (2022) 99:105299. doi: 10.1016/j.jff.2022.105299

[34] Javanmanesh F Kashanian M Rahimi M Sheikhansari N. A comparison between the effects of metformin and N-acetyl cysteine (NAC) on some metabolic and endocrine characteristics of women with polycystic ovary syndrome. Gynecol Endocrinol. (2016) 32:285–9. doi: 10.3109/09513590.2015.1115974, PMID: 26654154

[35] De Oliveira CPMS Stefano JT De Siqueira ERF Silva LS de Campos Mazo DF Lima VMR. Combination of N-acetylcysteine and metformin improves histological steatosis and fibrosis in patients with non-alcoholic steatohepatitis. Hepatol Res. (2008) 38:159–65. doi: 10.1111/j.1872-034X.2007.00215.x, PMID: 18197877

[36] Zarei M Zarezadeh M Khademi F Adeli S Abbaszade F Nikpayam O. What are the effects of N-acetylcysteine supplementation on anthropometric indices? A systematic review and meta-analysis of clinical trials. PharmaNutrition. (2020) 14:100238. doi: 10.1016/j.phanu.2020.100238

[37] Karczewski J Śledzińska E Baturo A Jończyk I Maleszko A Samborski P. Obesity and inflammation. Eur Cytokine Netw. (2018) 29:83–94. doi: 10.1684/ecn.2018.0415, PMID: 30547890

[38] Arulselvan P Fard MT Tan WS Gothai S Fakurazi S Norhaizan ME. Role of antioxidants and natural products in inflammation. Oxidative Med Cell Longev. (2016) 2016:1–15. doi: 10.1155/2016/5276130, PMID: 27803762 PMC5075620

[39] Biran A Zada L Abou Karam P Vadai E Roitman L Ovadya Y. Quantitative identification of senescent cells in aging and disease. Aging Cell. (2017) 16:661–71. doi: 10.1111/acel.12592, PMID: 28455874 PMC5506427

