# Supplementary material for: Effects of N-acetylcysteine on aging cell and obesity complications in obese adults: a randomized, double-blind clinical trial
Source: Front Nutr. 2023 Sep 19;10:1237869. doi: 10.3389/fnut.2023.1237869 (PMC10546057; doi:10.3389/fnut.2023.1237869)
Supplement: Supplementary file 1 [file Table_1.docx]

***Table S1: List of human primer sequences***

| ***Human gene*** | ***Forward*** | ***Reverse*** |
| --- | --- | --- |
| P16 | AAGCCATTGCGAGAACTT | CAGAGGGCAGAAAGAAAA |
| IL-6 | GGTACATCCTCGACGGCATCT | GTGCCTCTTTGCTGCTTTGAC |
| TNF-α | GCTCCAGACGGTGCTTGTG | GCCGATCACCCCAAAGTG |
